# Identification and Application of Neutralizing Epitopes of Human Adenovirus Type 55 Hexon Protein

**DOI:** 10.3390/v7102896

**Published:** 2015-10-27

**Authors:** Xingui Tian, Qiang Ma, Zaixue Jiang, Junfeng Huang, Qian Liu, Xiaomei Lu, Qingming Luo, Rong Zhou

**Affiliations:** 1 State Key Laboratory of Respiratory Disease, Guangzhou Institute of Respiratory Disease, The First Affiliated Hospital of Guangzhou Medical University, Guangzhou Medical University, Guangzhou 510230, China; tianxingui7902@aliyun.com (X.T.); mat008@163.com (Q.M.); 2 Dongguan Institute of Pediatrics, Dongguan Children’s Hospital, Dongguan 523325, China; jiangzaixue160368@126.com (Z.J.); qianliu_ln@163.com (Q.L.); lxm020@126.com (X.L.); 3 School of Life Sciences, Sun Yat-sen University, Guangzhou 510275, China; jfhuang.dg@gmail.com

**Keywords:** adenovirus type 55, neutralizing epitope, bivalent vaccine

## Abstract

Human adenovirus type 55 (HAdV55) is a newly identified re-emergent acute respiratory disease (ARD) pathogen with a proposed recombination of hexon gene between HAdV11 and HAdV14 strains. The identification of the neutralizing epitopes is important for the surveillance and vaccine development against HAdV55 infection. In this study, four type-specific epitope peptides of HAdV55 hexon protein, A55R1 (residues 138 to 152), A55R2 (residues 179 to 187), A55R4 (residues 247 to 259) and A55R7 (residues 429 to 443), were predicted by multiple sequence alignment and homology modeling methods, and then confirmed with synthetic peptides by enzyme-linked immunosorbent assay (ELISA) and neutralization tests (NT). Finally, the A55R2 was incorporated into human adenoviruses 3 (HAdV3) and a chimeric adenovirus rAd3A55R2 was successfully obtained. The chimeric rAd3A55R2 could induce neutralizing antibodies against both HAdV3 and HAdV55. This current study will contribute to the development of novel adenovirus vaccine candidate and adenovirus structural analysis.

## 1. Introduction

Human adenovirus (HAdV) has been recognized as a common cause of acute respiratory disease (ARD) [1,2,3]. To date, there are at least 69 HAdV genotypes reported [4], which are classified within seven species using a new paradigm based on genomics [5]. Among these, HAdV55 is a newly identified re-emergent acute respiratory disease (ARD) pathogen causing outbreaks in Singapore in 2005 and in Shanxi Province of China in 2006. After this first reported HAdVB55-associated ARD outbreak in China, this pathogen apparently re-emerged among military and civilian populations in many provinces of China [6,7,8,9,10]. HAdV55 infection causes both mild and severe diseases, presenting clinical signs and symptoms including high fever, cough, myalgia, sore throat, bronchitis and pneumonia, and even life-threatening [6,7,8,9,10,11,12,13]. Furthermore, HAdV55 has the potential to spread widely and cause severe epidemics in view of the lack of herd immunity and its higher tendency in causing severe ARD than other adenoviruses [7]. This status highlights the need for vaccine development against HAdV55 infection. HAdV55 was first identified as HAdV-B11a from an outbreak in a military trainee in Spain in 1969 [14,15,16]. Bioinformatics analysis based on genome sequences demonstrated that HAdV55 evolved from an intertypic recombinantion event in the hexon gene between HAdV-B11 and HAdV-B14 of the same subspecies B2, containing the major hexon gene from a renal pathogen, HAdV-B11, and other parts from a respiratory pathogen, HAdV-B14 [15,16,17,18,19,20,21,22].

The adenovirus capsid icosahedron is composed of three major structural proteins: hexon, penton base, and fiber. The hexon protein is the major antigenic determinant recognized by neutralizing antibodies (NAbs) [23,24,25,26]. It is important to identify neutralizing epitopes in designing novel vaccines, developing antiviral drugs and rapid diagnostic reagents. Type-specific epitopes on hexons have been proposed to reside within seven highervariable regions (HVRs) by multiple sequence alignment and homology modeling methods [27,28,29,30]. We and other groups have identified neutralizing epitopes of few serotypes including HAdV3, HAdV7, AdC68, and HAdV5 [31,32,33,34,35,36,37]. However, up to now, no neutralizing epitope has been identified for other HAdVs including HAdV55.

The current study sought to map the neutralizing epitopes of HAdV55. Furthermore, we tried to incorporate the epitopes on the surface of HAdV-3 hexon to prepare a novel bivalent vaccine candidate against both HAdV3 and HAdV55. This study expands current knowledge about HAdV and will be useful for HAdV vaccine development.

## 2. Results

### 2.1. Identification of Neutralizing Epitopes Using Synthetic Peptide

**Figure 1:**
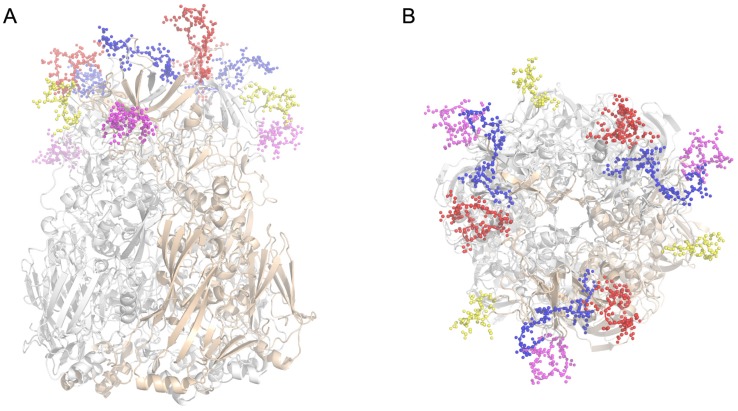

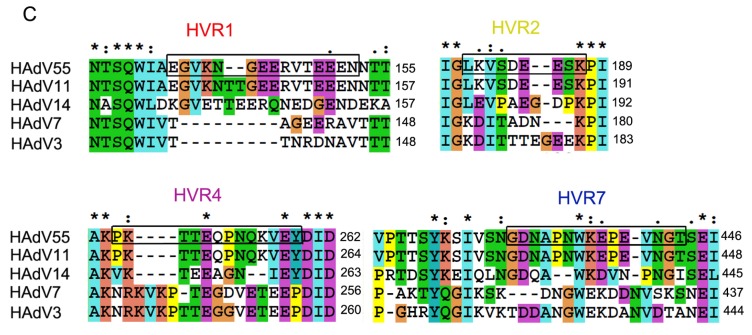
Defining the potential neutralizing epitopes of HAdV55 hexon. (**A**,**B**) 3D model of HAdV55 hexon and the predicted NAb epitopes in the tower regions (**A**: side view, **B**: top view). A55R1, A55R2, A55R4 and A55R7 were colored by red, yellow, magentas and blue, respectively; (**C**) Amino acid sequence alignment of several HAdV species B hexons. Only four hypervariable regions (HVRs) are shown. The number shows the position of the right side amino acid in the corresponding hexon protein. *, conserved amino acid; **:**, either size or hydropathy is conserved; **.**, and both size and hydropathy are conserved. Gaps used to optimize alignments are indicated by dashes. The corresponding amino acid sequences of A55R1, A55R2, A55R4 and A55R7 are marked in boxes.

Previous studies suggest that human adenovirus serotype-specific NAb epitopes may be exposed on the virion surface and reside within any of the seven hypervariable regions (HVRs) [27,30,31,33]. Using sequence alignment data and the 3D structure of HAdV55 hexon by homology modeling using the known crystal structure of AdC68 hexon (PDB_ID 2OBE) as a template, four regions within the HAdV55 hexon (A55R1, residues 138EGVKNGEERVTEEEN152; A55R2, residues 179LKVSDEESK187; A55R4, residues 247PKTTEQPNQKVEY259; and A55R7, residues 429GDNAPNWKEPEVNGT443) were predicted as candidate neutralizing epitopes, which are all located in the tower region that stretch to the external environment (Figure 1A,B). Sequence alignment between the HAdV55 and HAdV11 hexon proteins demonstrated variations of amino acids only in HVR1 (Figure 1C).

**Figure 2:**
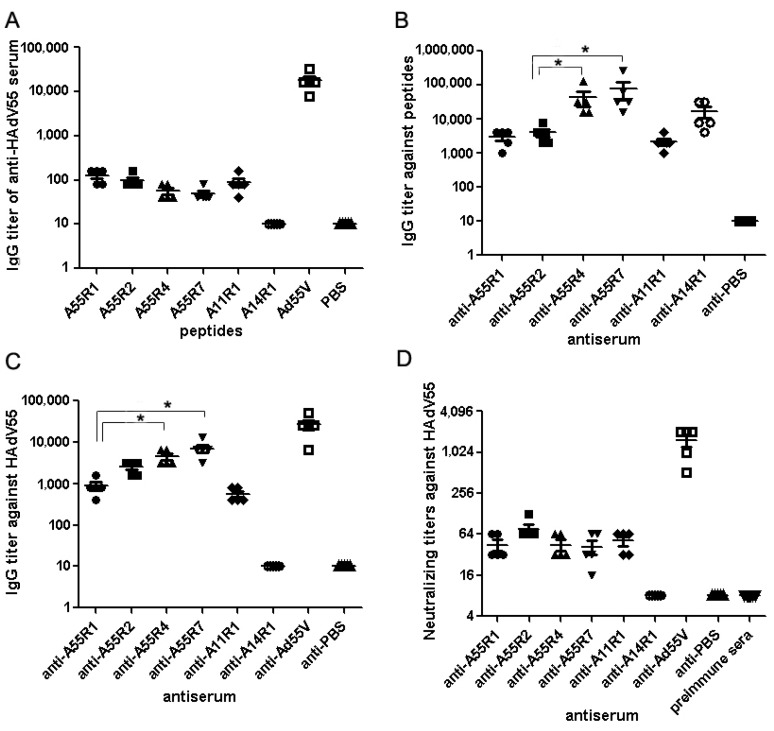
Identification of NAb epitopes of HAdV55 hexon with synthetic peptides. (**A**) Indirect ELISAs were performed to access the reactions of anti-HAdV55 sera with predicted synthetic peptides. The synthetic peptide of A14R1 (ETTEERQNEDGENDEKA) and PBS was used as a negative control, purified HAdV55 virions was used as the positive control; (**B**) ELISAs of the anti-peptide sera with synthetic peptides; (**C**) ELISAs of the anti-peptide sera with purified HAdV55 virus particles. The antisera from mice immunized with PBS and A14R1 were used as the negative controls; (**D**) *In vitro* neutralization tests. Neutralization tests were performed with the anti-peptide sera against HAdV55. The anti-PBS and anti-A14R1 sera were used as the negative controls. Each experiment was repeated independently at least three times. Each symbol represented a mouse, and the lines indicated the means or means ± SEM for each group of mice. * *p* < 0.05.

To test the antigenicity of the predicted neutralizing epitopes, ELISAs were performed by coating synthetic peptides and reacting with anti-HAdV55 sera. The synthetic peptide A14R1 (ETTEERQNEDGENDEKA) and A11R1 (EGVKNTTGEERV), and the purified HAdV55 virions were used as the controls. Figure 2A shows that the four putative peptides can bind to the anti-HAdV55 sera but at a weak titer.

Furthermore, antibody responses of mice immunized with KLH-coupled peptides were also measured by ELISAs. Antibody titers were detected by ELISA using synthetic peptides, which differed from 1:2000 to 1:256,000 (Figure 2B). ELISA analysis with purified HAdV55 virions showed that IgG titers of anti-A55R4 and anti-A55R7 groups sera were significantly higher than that of anti-A55R1 and anti-A55R2 groups sera, which were coincident with the result of ELISA with synthetic peptides (Figure 2C). Finally, *in vitro* neutralization tests were performed with serially diluted anti-peptides sera (anti-A55R1, anti-A55R2, anti-A55R4, anti-A55R7, anti-A11R1 and anti-A14R1), anti-PBS and anti-HAdV55 sera, neutralizing HAdV55 cultured in AD293 cells. After continuous observation for 72 h, the anti-A55R1, anti-A55R2, anti-A55R4, anti-A55R7, anti-A11R1, and anti-HAdV55 sera could neutralize HAdV55 infection; whereas, the anti-A14R1, anti-PBS and all preimmune sera could not neutralize HAdV55 infection, even at the lowest dilution, 1:8 (Figure 2D). The NT results indicated the four residues as neutralizing epitopes.

**Figure 3:**
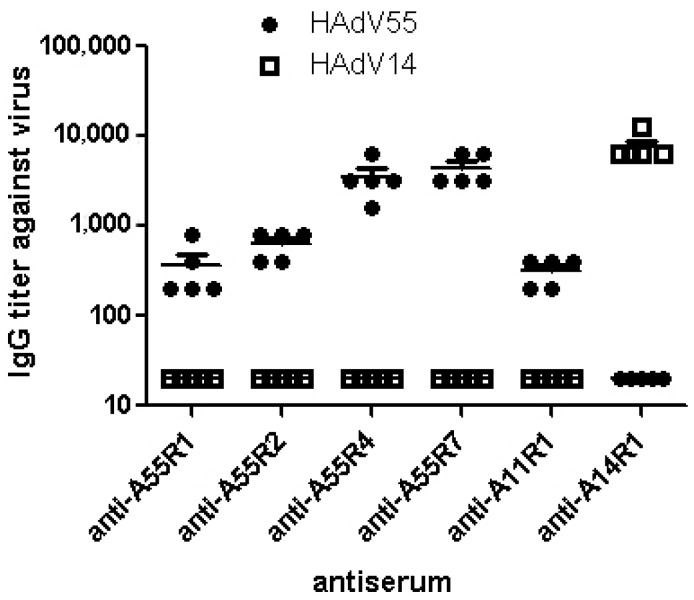
The type-specificity of anti-peptide sera by ELISA. Indirect ELISAs were performed to access the crossreactions of anti-peptide sera with purified HAdV55 and HAdV14 virions. Each experiment was repeated independently at least three times. Each symbol represented a mouse, and the means ± SEM for each group of mice are shown with the lines.

Furthermore, ELISA demonstrated that anti-A55R1, A55R2, A55R4 and A55R7 sera could only detect HAdV55 but not HAdV14 virions. In contrast, anti-A14R1 sera could only detect HAdV14 but not HAdV55 virions (Figure 3). These results indicate these four epitopes are serotype-specific. It is interesting to find that anti-A11R1 sera could detect HAdV55 but not HAdV14 virions, which is coincident with the NT results.

### 2.2. Anti-rAd3A55R2 Serum Could Neutralize Both HAdV3 and HAdV55 in Vitro

**Figure 4:**
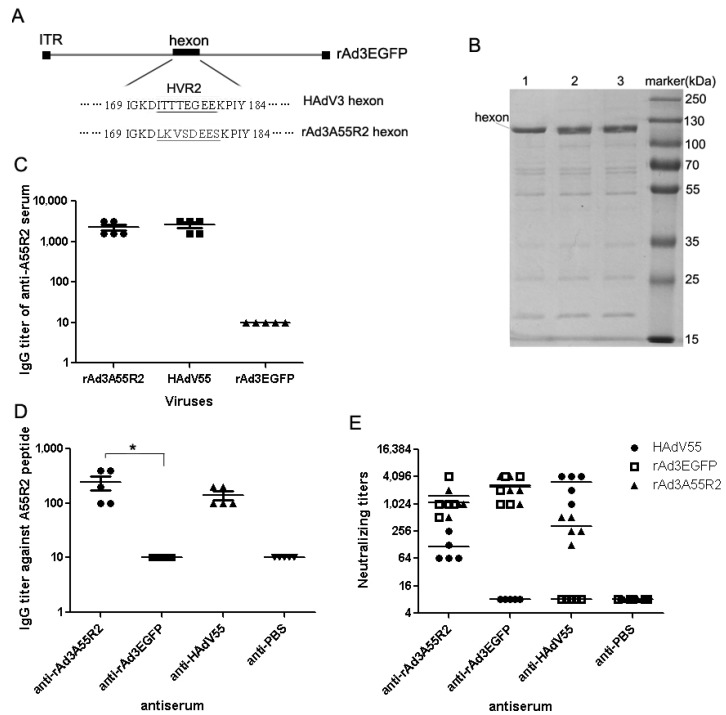
The antigenicity of the epitope chimeric recombinant rAd3A55R2. (**A**) Schematic depiction of rAd3A55R2 replacing HVR2 of rAd3EGFP with A55R2. HVR2 amino acid sequences of HAdV3 and HVR2 amino acid sequences of HAdV55 incorporated in rAd3A55R2 hexon are shown with underline; (**B**) Recombinant rAd3A55R2 confirmation by SDS-PAGE, **1**: HAdV55, **2**: rAd3A55R2, and **3**: rAd3EGFP virions; (**C**) ELISA with anti-A55R2 sera as primary antibody with purified rAd3A55R2 and HAdV55, rAd3EGFP virions; (**D**) ELISAs of the anti-rAd3A55R2 sera with synthetic peptide A55R2. The anti-HAdV55 and anti-rAd3EGFP were used as the controls; (**E**) *In vitro* neutralization tests. Neutralization tests were performed with the anti-rAd3A55R2 sera against HAdV55 and rAd3EGFP. The anti-HAdV55 and anti-rAd3EGFP sera were used as the controls. Each experiment was repeated independently at least three times. Each symbol represented a mouse, and the lines indicated the means or means ± SEM. * *p* < 0.05.

In the current study, all four putative epitopes were tried to incorporated into the corresponding HVRs of HAdV-3, however, only the chimeric adenovirus rAd3A55R2 was successfully rescued, amplified and subsequently purified by CsCl centrifugation (Figure 4A). Repeated attempts to rescue and amplify the other three chimeric Ads were unsuccessful. The hexon modification of viruses of rAd3A55R2 was confirmed by PCR and sequencing using genomic DNA from the purified virions. The purified rAd3A55R2 were confirmed by SDS-PAGE (Figure 4B) and indirect ELISA with anti-A55R2 sera.

To verify the immunizing potential of this chimeric virus against both HAdV3 and HAdV55, anti-rAd3A55R2 sera from mice were characterized by ELISA and *in vitro* neutralizing test. As shown in Figure 4D, rAd3A55R2 immunization in mouse could induce A55R2-specific antibody response. As shown in Figure 4E, anti-rAd3A55R2 sera could neutralize HAdV55 infection with titers ranging from 1:2^6^ to 1:2^8^, and neutralize HAdV3 infection with titers ranging from 1:2^9^ to 1:2^12^. As controls, anti-AD3EGFP or anti-HAdV55 sera could not neutralize each other virus at the lowest dilution tested 1:2^4^.

## 3. Discussion

In this study, we predicted and verified four neutralizing epitopes within the HAdV55 hexon, of which A55R2 was successfully incorporated into HAdV3 to generate a bivalent vaccine candidate against HAdV3 and HAdV55.

It is important to determine neutralizing epitopes of pathogenic viruses for vaccine design, rapid diagnostic reagent and antiviral drug development. In the case of human adenoviruses including more than 69 known genotypes, although previous hexon crystallographic and phylogenetic analyses have suggested that serotype-specific neutralizing epitopes might reside in any of the seven HVRs exposed to the viral surface [26,27,28,29,30], only few epitopes have been identified so far [31,32,33,34]. In our previous studies, we identified several epitopes of HAdV-3 and HAdV-7 with a series of MAbs and epitope-incorporated recombinant adenoviruses [34,35,36]. In the current study, by defining the 3D confirmation of HAdV55 hexon by homology modeling to determine the surface exposing regions, in combination with MSA of species B hexons to detect serotype-specific residues [30,31,32,33,34], four serotype-specific neutralizing epitopes were predicted. These four type-specific neutralizing epitopes of HAdV55 hexon protein were then mapped accurately by peptide ELISAs and NTs. The therapeutic neutralizing monoclonal antibodies and preventive novel vaccines against HAdV55 based on these epitopes could be developed. However, the current study did not confirm whether the other HVRs and other parts of hexon contain neutralizing epitopes could be revealed in the further study. In any case, the current study partially validated the quick prediction method of neutralizing epitopes in adenovirus hexon.

The four putative peptides can bind to the anti-HAdV55 sera but at weak titers (Figure 2A). The ELISA results using peptides and purified HAdV55 virion shown that KLH-coupled peptides A55R4 and A55R7 immunization in mice generated higher titer antibody responses than A55R1 and A55R2 (Figure 2B,C), but the NAb titers of anti-peptides sera were not significantly different (Figure 2D). These results could be explained by two reasons. First, the epitopes on HAdV55 virions are conformational in part. Second, although the KLH-coupled peptides A55R4 and A55R7 had stronger immunigenicity in mouse, but the neutralization activity may be affected by the epitope position, not only by the antibody titer.

HAdV55 was first incorrectly termed “HAdV11a” with a proposed recombination of hexon gene between HAdV11 and HAdV14 strains [14,15,16,17,18,19,20,21,22]. Previous study found antisera against HAdV55 could neutralize HAdV11 at a high titer, and neutralize HAdV14 at a lower titer [14,38]. Alignment between the amino acid sequences of hexon from HAdV55 and HAdV11 demonstrated that there are two continuous amino acids deleted in HVR1 of HAdV55 (Figure 1B). It is also found that anti-A11R1 could detect and neutralize HAdV55 by ELISA and NTs (Figure 2). So the deleted amino acids may be not the critical amino acids recognized by the serotype-specific neutralizing antibodies. Further ELISA of Figure 3 result demonstrated that these four epitopes are type-specific, of which anti-A55R4 and anti-A55R7 sera have stronger reaction with HAdV55. These results may be useful for developing HAdV55-specific diagnostic reagents.

In recent years, an increasing trend of HAdV55 infections were observed among both civilian and military populations, and HAdV55 could cause severe respiratory disease with a higher tendency than other adenoviruses [5,6,7,8,9,10,11,12,20,21]. It is necessary to develop licensed HAdV55 vaccine to prevent HAdV55 infection outbreaks. Our group has constructed a recombinant HAdV-3 vector named Ad3EGFP as a tool for viral delivery or live-vaccine construction, and a vaccine platform by “capsid-incorporation strategy” [39,40,41,42]. We also generated a bivalent vaccine candidate rAdMHE3 that elicited protective immunity to both HAdV-3 and HAdV-7 by replacing epitope R3 within HAdV3 hexon with HAdV-7 neutralizing epitope E3 [34]. In this paper, we sought to generate a bivalent vaccine against HAdV3 and HAdV55 using the same strategy. As a result, a chimeric adenovirus rAd3A55R2 was successfully generated that could induce neutralizing antibodies against both HAdV3 and HAdV55 (Figure 4). However, the NAbs titer against HAdV55 is much lower than that against HAdV3, thus it is necessary to further prepare chimeric adenoviruses that can induce balanced antibody responses against HAdV3 and HAdV55. In this study, the attempts to rescue and amplify chimeric Ads incorporated with the other three epitopes were unsuccessful. However, it is possible to prepare the chimeric Ads incorporated with the other three epitopes by optimizing the incorporated HAdV55 epitopes and the inserted sites of HAdV3 [43]. More practical chimeric adenoviruses might be obtained by this way. It is also necessary to identify the distribution of the epitope among HAdV55 strains that might affect the neutralization ability of the antibody [44,45]. By sequence alignment of the globally known HAdV55 and HAdV11 hexon sequences available in GenBank, we found the epitope in HVR2 of different HAdV55 and HAdV11 strains is highly conserved. The current findings contribute not only to the development of new adenovirus vaccine candidates, but also to adenovirus structure knowledge and the construction of new gene delivery vectors.

## 4. Materials and Methods

### 4.1. Virus Strains and Cells

The recombinant adenovirus rAd3EGFP encoding a HAdV-3 GZ-01 genome (Genbank accession no. DQ099432) and an enhanced green fluorescent protein (eGFP) with an E3 region deletion were obtained as previously described [39]. HAdV-14 GZ01 strain (GenBank no. JQ824845.1) was kindly provided by Qiwei Zhang of Sourthern Medical University (Guangzhou, China) [46]. HAdV55 Shanxi-Y16 strain (GenBank no. KF911353.1) was kindly provided by Lin Chen of Guangzhou Medical University (Guangzhou, China). All the adenoviruses were cultured in HEp-2 cells or AD293 cells, and adenovirus particles were purified by standard CsCl gradient centrifugation as previously described [23]. The virus particle (VP) titers were determined by spectrophotometry using a conversion factor of 1.1 × 10^12^ VPs per absorbance unit at 260 nm [23].

### 4.2. Defining the Potential NAb Epitopes of HAdV55 Hexon

The HVRs of HAdV55 hexon protein were determined by multiple sequence alignment (MSA) with the available hexon protein sequences of species B human adenoviruses using the protein BLAST program (Basic Local Alignment Tool) [47] or ClustalW 1.83 software using a progressive algorithm and adjusted manually. The following hexon protein sequences from Genbank are used: HAdV55(AHK61135.1), HAdV11(ACZ06784.1), HAdV14(AAZ99996.1), HAdV7(CAA88460.1), HAdV3(AAZ15255.1), HAdV11(YP_002213812), HAdV35(BAB20015), and HAdV21(AAG21823). The appropriate templates for the homology modeling were identified by searching them in the Protein Data Bank (PDB) using HAdV55 hexon as a probe [48]. The Modeler 9v8 tool was applied to model the three-dimensional (3D) structure [38]. The antigenic epitopes with length between 6 and 15 amino acids that were predicted to be exposed on the capsid surface and located in HVRs were selected as potential sites for recognition by NAbs [33].

### 4.3. Peptides Synthesis

The four predicted epitope peptides were synthesized by GL Biochem Ltd. (Shanghai, China), and then purified and analyzed by highperformance liquid chromatography (purity ≥90%). The Cys was added on the N-terminal of synthetic peptides for conjugation. Then, each peptide was chemically linked to the carrier protein keyhole limpet hemocyanin (KLH). Meanwhile, two control peptides (A14R1 from HAdV14 and A11R1 from HAdV11) were synthesized by the same method.

### 4.4. Generating of Epitope Chimeric Adenoviruses

The plasmid pBRAdΔE3GFP encoding a HAdV-3 GZ-01 genome and eGFP with an E3 region deletion and the shuttle vector pBRLR was previously constructed by us [24,33]. In this study, the chimeric mutant rAd3A55R2 containing putative HAdV55 epitope A55R2 in HVR2 of HAdV-3 was obtained using the same strategy as our previously described [33].

Briefly, the mutated fragment H3A55R2 was produced by overlapping PCR extension mutagenesis, and then cloned into pBRLR to generate shuttle vectors pBRLR-H3A55R2. Finally, the LR-H3A55R2 fragment was cloned into the pBRAdΔE3GFP vector to generate the HAdV-3 hexon HVR2 mutagenesis vector pBRAd3-A55R2 using homologous recombinant technology in *Escherichia coli* (*E. coli*) strain BJ5183. The successful creation of the constructs was confirmed by restriction digestion and sequencing analyses.

To rescue viruses, these modified plasmids were digested with AsisI to linearize genomic DNA, then transfected into AD293 cells grown in 30-mm dishes using Lipofectamine LTX with Plus reagents (Invitrogen, Carlsbad, CA, USA) according to the manufacturer’s instructions. The transfected cells were cultured at 37 °C with 5% CO_2_ for 6–10 days and were examined daily for evidence of cytopathic effect. The virus was harvested and designated rAd3A55R2. Finally, the mutant virus was cultured with AD293 cells in a total of twenty 100 mM dishes, then harvested and purified by standard CsCl gradient centrifugation as described above. The full-length modified hexon genes of the viruses were identified by sequencing.

### 4.5. Mouse Immunization

Female Balb/c mice aged 4–6 weeks were used in the immunization experiments. Mice in groups of 5 were injected intramuscularly (i.m.) with 5 × 10^9^ VPs per mouse (about 10 μg total protein) rAd3A55R2, rAd3EGFP and inactivated HADV55 (inactivated with 20 mmol/L beta-propiolactone at 4 °C for 24 h) viron, or 50 μg KLH-conjugated peptides in QuickAntibody-Mouse5W adjuvant (Biodragon Immunotechnologies Ltd., Beijing, China), followed by one additional boost 3 weeks later with the same doses and method. Phosphate buffered saline (PBS) in the same adjuvant was injected to mouse as the control. Blood samples were collected from the mice at two weeks after the final immunization and sera were isolated, heat-inactivated and kept frozen for serology tests. The animal procedures used in this work were evaluated and approved by the Ethic Committee of the First Affiliated Hospital of Guangzhou Medical University (project no. 31373303, 11/3/2013). They complied with all relevant guidelines and the National law for Laboratory Animal Experimentation. The animal experiments were conducted in strict accordance with the recommendations of the Guide for the Care and Use of Laboratory Animals of the National Institutes of Health. All animals were housed in individually and received humane care. During injection and sample collection, the mice were anesthetized with 1.5% isoflurane or 1mL/kg weight 3% pentobarbital sodium to minimize suffering. 

### 4.6. ELISA

For ELISAs, the ELISA plates (Corning Inc., New York, NY, USA) were coated overnight at 4 °C with synthetic peptides (about 2 µg/mL) or purified virus particles (about 10^10^ VPs/mL) in carbonate–bicarbonate buffer (pH 9.6), and were washed once with 0.05% Tween-20 in phosphate-buffered saline (PBST) and blocked for 2 h with 5% skim milk in PBST. Then antisera of adenoviruses or peptides serially diluted in PBS were added and incubated for 1 h at 37 °C. After being washed three times with PBST, the plates were added with a 1:8000 dilution of HRP-Conjugated goat anti-mouse IgG (H + L) secondary antibody (CWBio Inc., Beijing, China) and incubated for 1 h. After washing four times with PBST, the plates were developed with tetramethylbenzidine (TMB) substrate, stopped with 2 M H_2_SO_4_, and analyzed at 450 nm using an ELISA plate reader (Thermo Scientific Multiskan MK3, Shanghai, China). Endpoint titer was defined as the highest dilution at which the OD_450_ was at least 0.1 above wells receiving no sera. Wells receiving no sera always had an OD_450_ of <0.1. Each assay was performed independently at least three times and with at least two parallel reactions for each well.

### 4.7. Neutralization Tests

NTs were performed to test the neutralizing effect of antisera to HAdV55 or HAdV3. First, sera were heated at 56 °C for 30 min to inactivate complement. The half tissue culture infective dose (TCID50) of purified adenoviruses were detected by the typic method with AD293 cells. All of the antipeptides sera and anti-rAd3A55R2, anti-PBS sera as a negative control, and anti-HAdV55, anti-rAd3EGFP sera as positive controls, were serially 2-fold diluted (1:8 to 1:16,384) in DMEM, and 50 µL aliquots of each dilution were mixed with 50 µL of virus with 100 TCID50. The antibody-virus mixtures were incubated at 37 °C for 1 h and transferred to 96-well plates containing 70%–90% confluent monolayers of AD293 cells. The monolayers were cultured for 96 h, after which the infection was observed by microscopy and the neutralization titers were determined as the reciprocal of the highest serum dilution that completely inhibited visually observable cytopathic effect (CPE).

### 4.8. Statistical Analyses

Statistical significance was determined using Prism 5.0 software (GraphPad Prism Inc., La Jolla, CA, USA). Comparisons between two groups were made with Student’s *t* tests. Comparisons among groups were performed by ANOVA with Bonferroni’s test to account for multiple comparisons and *p* values of less than 0.05 were considered statistically significant.
